# Impact of the Coronavirus disease pandemic on early breast cancer

**DOI:** 10.3389/fonc.2024.1412027

**Published:** 2024-11-27

**Authors:** Yong Li, Xiao-ju Lu, Bo Xu, Wei-wen Li

**Affiliations:** ^1^ Department of Breast, Jiangmen Central Hospital, Jiangmen, China; ^2^ Department of General Surgery, The First Affiliated Hospital of Jinan University, Guangzhou, China; ^3^ Department of Critical Care Medicine, Jiangmen People’s Hospital, Jiangmen People’s Hospital, Jiangmen, China; ^4^ Department of General Surgery, Guangzhou First People’s Hospital, School of Medicine, South China University of Technology, Guangzhou, China

**Keywords:** COVID-19 pandemic, overall survival, cancer diagnosis, breast cancer, cancer treatment

## Abstract

**Objective:**

To assess the impact of the COVID-19 pandemic on the presentation, treatment, and survival of patients with early breast cancer (Stage I–III).

**Methods:**

This study utilized data from the Surveillance, Epidemiology, and End Results database from January 1, 2018, to December 31, 2020. Patients diagnosed with primary breast cancer in 2020 were compared to those diagnosed in 2018 or 2019. The primary outcomes were stage distribution and changes in the treatment modalities for early breast cancer. The secondary outcomes were overall survival (OS) and breast cancer-specific survival (BCSS).

**Results:**

We analyzed 142,038 patients. There has been a decrease in breast cancer diagnoses in 2020, as well as the smaller number of surgeries. The distribution of breast cancer stages among patients exhibited a notable shift in 2020, with a decrease in the proportion of Stage 0-I and an increase in advanced-stage. Additionally, there was a significant decrease in the proportion of breast-conserving surgery (BCS) performed in 2020. The proportion of patients undergoing radiation decreased, while that of chemotherapy cases increased significantly in 2020. Patients showed a shorter treatment delay in 2020 than in 2018 or 2019 (2018: hazard ratio [HR] = 0.969, 95% confidence interval [CI] = 0.956–0.982, *p* < 0.001; 2019: HR=0.959, 95% CI = 0.946–0.972, *p* < 0.001). Diagnosis in 2020 showed a significant correlation with worse OS than diagnosis in 2018 (HR = 0.861, 95% CI = 0.743-0.996, *p* = 0.045).

**Conclusion:**

We observed a shift to advanced-stage and a change of treatment modalities of early breast cancer in 2020. The OS of patients with breast cancer was worse during the pandemic than before the pandemic. The findings could provide empirical basis for optimizing cancer prevention and control strategies in future public health emergencies.

## 1 Introduction

COVID-19 triggers SARS by inflaming the respiratory tract and activating immune cells to produce excessive pro-inflammatory cytokines (1). By the beginning of 2023, the World Health Organization reported approximately 750 000 000 confirmed cases of COVID-19 and more than 6 800 000 deaths. The Covid-19 pandemic spread worldwide and place a significant strain on the healthcare systems.

Breast cancer screenings were largely disrupted during the onset of the COVID-19 pandemic (2–4), leading to a decrease in the number of diagnosed cases in 2020 (5–7). Additionally, socioeconomic disparities may have aggravated this risk (8, 9). Various studies have shown that the pandemic led to patients presenting with advanced stages of breast cancer (10, 11). However, Tonneson et al. published contrasting findings that the breast cancer stage at diagnosis did not differ significantly between the COVID-19 and pre-COVID-19 periods (12). The study from the Netherlands Cancer Registry supported these conclusions (5). So far, it is unclear whether COVID-19 affected the stage of breast cancer. Meanwhile, few studies reported the effect of the pandemic on the treatment changes and prognosis of breast cancer.

This study aimed to investigate whether the COVID-19 pandemic affected the presentation, treatment and survival of patients with early breast cancer (Stage I–III).

## 2 Materials and methods

### 2.1 Study participants

The Surveillance, Epidemiology, and End Results (SEER) database is a systematic population-based cancer registry covering approximately 48% of the United States population. Clinically relevant data of patients diagnosed with breast cancer from 2018 to 2020 were extracted from the SEER 18 registry database (1975–2020) using SEER*Stat 8.4.2. STROCSS criteria was followed in this work (13). The need for ethical approval was waived because the SEER is a publicly accessible and freely available database. The year 2020 marks the onset of the COVID-19 pandemic, and we utilized data from 2018 and 2019 as control data. Patients with primary breast cancer were included in the study. The following variables were extracted: patient ID, race, age, grade, stage as per the American Joint Committee on Cancer (8th edition), subtype, radiation, chemotherapy, surgery code, vital status, cause of death, time to treatment initiation (TTI), and survival in months. Patients with unclear tumor grade or stage were excluded.

### 2.2 Outcomes of interest

The primary outcomes were cancer stage distribution, changes in the treatment modalities in early breast cancer, and treatment delay. Treatment modalities included surgery, radiation, and chemotherapy. Treatment delay was measured according to the TTI. The secondary outcomes were overall survival (OS) and breast cancer-specific survival (BCSS) in patients with Stage I–III tumors. OS was defined as the time from diagnosis to death or the last follow-up. BCSS was defined as the time from cancer to death due to breast cancer.

### 2.3 Statistical analyses

Patient demographics, tumor characteristics, and treatment information were compared among the patients diagnosed in 2018, 2019, and 2020 using the chi-square test with Bonferroni correction. Time-to-event analysis was performed to evaluate the TTI recorded. The multivariable Cox proportional hazards model was applied to evaluate the covariates associated with OS and BCSS. Considering the different follow-up durations among patients diagnosed in 2020 and 2018 or 2019, we used the modified Cox proportional hazards model. Patients were classified as “alive,” if they had not yet died 1 year after diagnosis. These results were presented as hazard ratios (HRs) with 95% confidence intervals (CIs). P-values <0.05 were considered statistically significant, and all tests were two-sided. All statistical analyses were performed using Statistical Product and Service Solutions (version 22.0; IBM, Armonk, NY, USA).

## 3 Results

**Figure 1:**
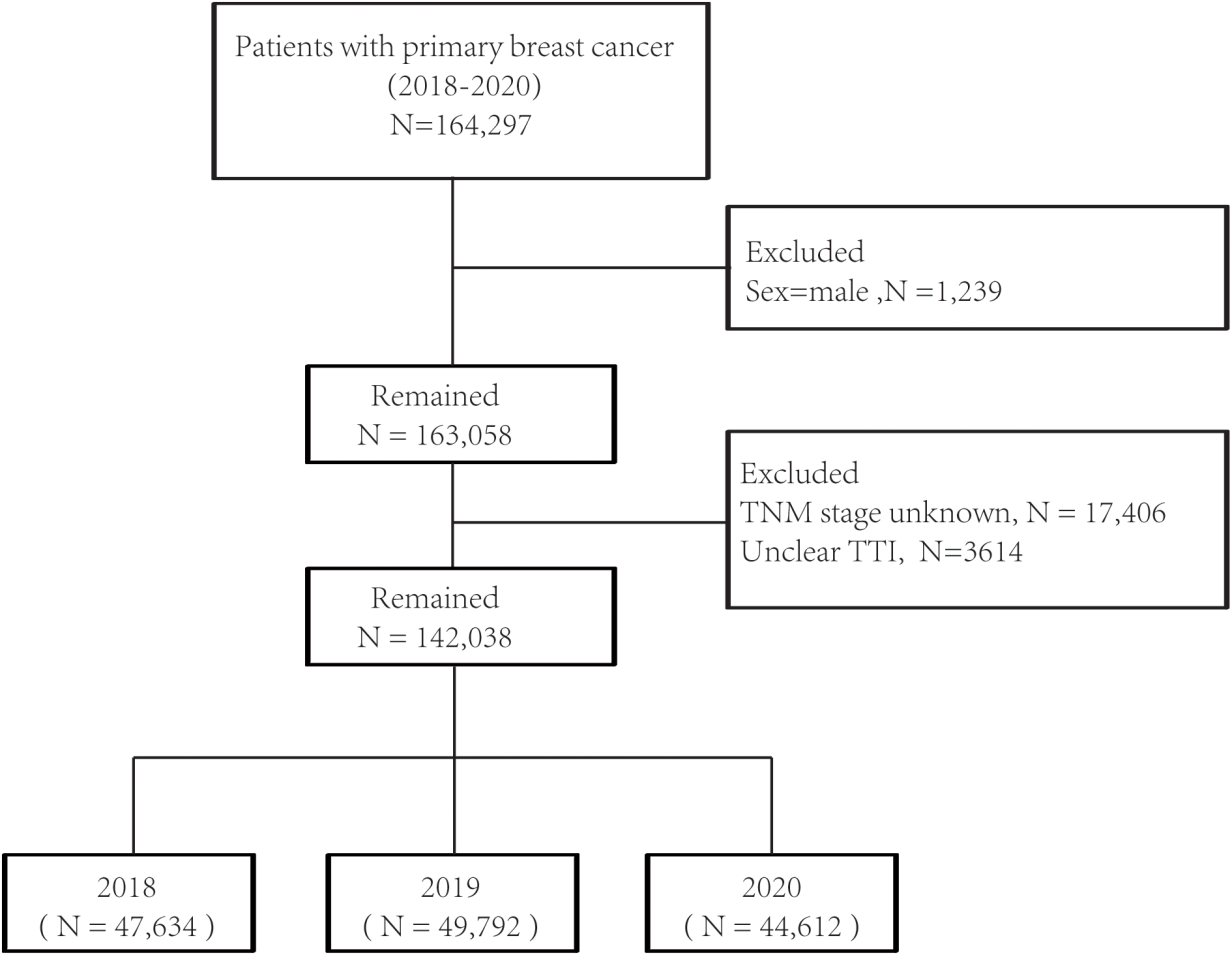
Flowchart for the SEER (Surveillance, Epidemiology, and End Results) data screening.

In total, 142,038 patients diagnosed with primary breast cancer between 2018 and 2020 were included in the final analysis, of which 47,634 (33.5%) were diagnosed in 2018, 49792 (35.1%) in 2019, and 44612 (31.4%) in 2020 (**Figure 1**). The median follow-up duration was 29 months (interquartile range, 25–32) in 2018 and 17 months (interquartile range, 14–20) in 2019, and it decreased to 5 months (interquartile range, 2–9) in 2020. As shown in **Table 1**, patients diagnosed with breast cancer in 2020 tended to be younger than those diagnosed in 2018 or 2019. Fewer patients diagnosed in 2020 underwent surgery and radiation but more patients received chemotherapy as compared to those diagnosed in 2018 or 2019. Moreover, treatment initiation in one month was more common among patients diagnosed in 2020 than those diagnosed in previous years.

**Table 1:** Demographics and clinical characteristics of patients diagnosed in 2018, 2019 and 2020.

Characteristics		2018 (n=47634)	2019 (n=49792)	2020 (n=44612)	Total (n=142038)	*P* value
Race						<0.001
	White	36610 (76.9%)	38177 (76.7%)	33944 (76.1%)	108731	
	Black	5104 (10.7%)	5452 (10.9%)	5016 (11.2%)	15572	
	Other	5479 (11.5%)	5728 (11.5%)	5120 (11.5%)	16327	
	Unknown	441 (0.9%)	435 (0.9%)	532 (1.2%)	1408	
Age						<0.001
	<50	9915 (20.8%)	10382 (20.9%)	9806 (22%)	30103	
	50≤;<70	24514 (51.5%)	25513 (51.2%)	22559 (50.6%)	72586	
	≥70	13205 (27.7%)_	13897 (27.9%)	12247 (27.5%)	39349	
Grade						<0.001
	I	10843 (22.8%)	11200 (22.5%)	9394 (21.1%)	31437	
	II	21629 (45.4%)	23182 (46.6%)	20786 (46.6%)	65597	
	III	14500 (30.4%)	14729 (29.6%)	13779 (30.9%)	43008	
	Unknown	662 (1.4%)	681 (1.4%)	653 (1.5%)	1996	
Subtype						<0.001
	HR+/Her2-	35728 (75%)	38055 (76.4%)	33422 (74.9%)	107205	
	HR+/Her2+	4945 (10.4%)	4778 (9.6%)	4526 (10.1%)	14249	
	HR-/Her2+	1899 (4%)	1804 (3.6%)	1796 (4%)	5499	
	HR-/Her2-	4872 (10.2%)	4931 (9.9%)	4673 (10.5%)	14476	
	Unknown	190 (0.4%)	224 (0.4%)	195 (0.4%)	609	
Stage						<0.001
	0	33 (0.1%)	42 (0.1%)	52 (0.1%)	127	
	I	34542 (72.5%)	36723 (73.8%)	32049 (71.8%)	103314	
	II	6933 (14.6%)	6773 (13.6%)	6432 (14.4%)	20138	
	III	3459 (7.3%)	3539 (7.1%)	3431 (7.7%)	10429	
	IV	2667 (5.6%)	2715 (5.5%)	2648 (5.9%)	8030	
Surgery						<0.001
	Aspiration/biopsy only	3087 (6.5%)	3535 (7.1%)	3755 (8.4%)	10377	
	Mastectomy	16578 (34.8%)	16734 (33.6%)	15268 (34.2%)	48580	
	Breast conserving	27944 (58.7%)	29465 (59.2%)	25554 (57.3%)	82963	
	Unknown	25 (0.1%)	58 (0.1%)	35 (0.1%)	118	
Radiation						<0.001
	No	19363 (40.6%)	20700 (41.6%)	19526 (43.8%)	59589	
	Yes	28271 (59.4%)	29092 (58.4%)	25086 (56.2%)	82449	
Chemotherapy						<0.001
	No	28797 (60.5%)	30675 (61.6%)	26258 (58.9%)	85730	
	Yes	18837 (39.5%)	19117 (38.4%)	18354 (41.1%)	56308	
Time to treatment initiation (month)						<0.001
	≤1	32374 (68%)	33091 (66.5%)	31063 (69.6%)	96528	
	>1	15260 (32%)	16701 (33.5%)	13549 (30.4%)	45510	

### 3.1 Stage distribution

**Figure 2:**
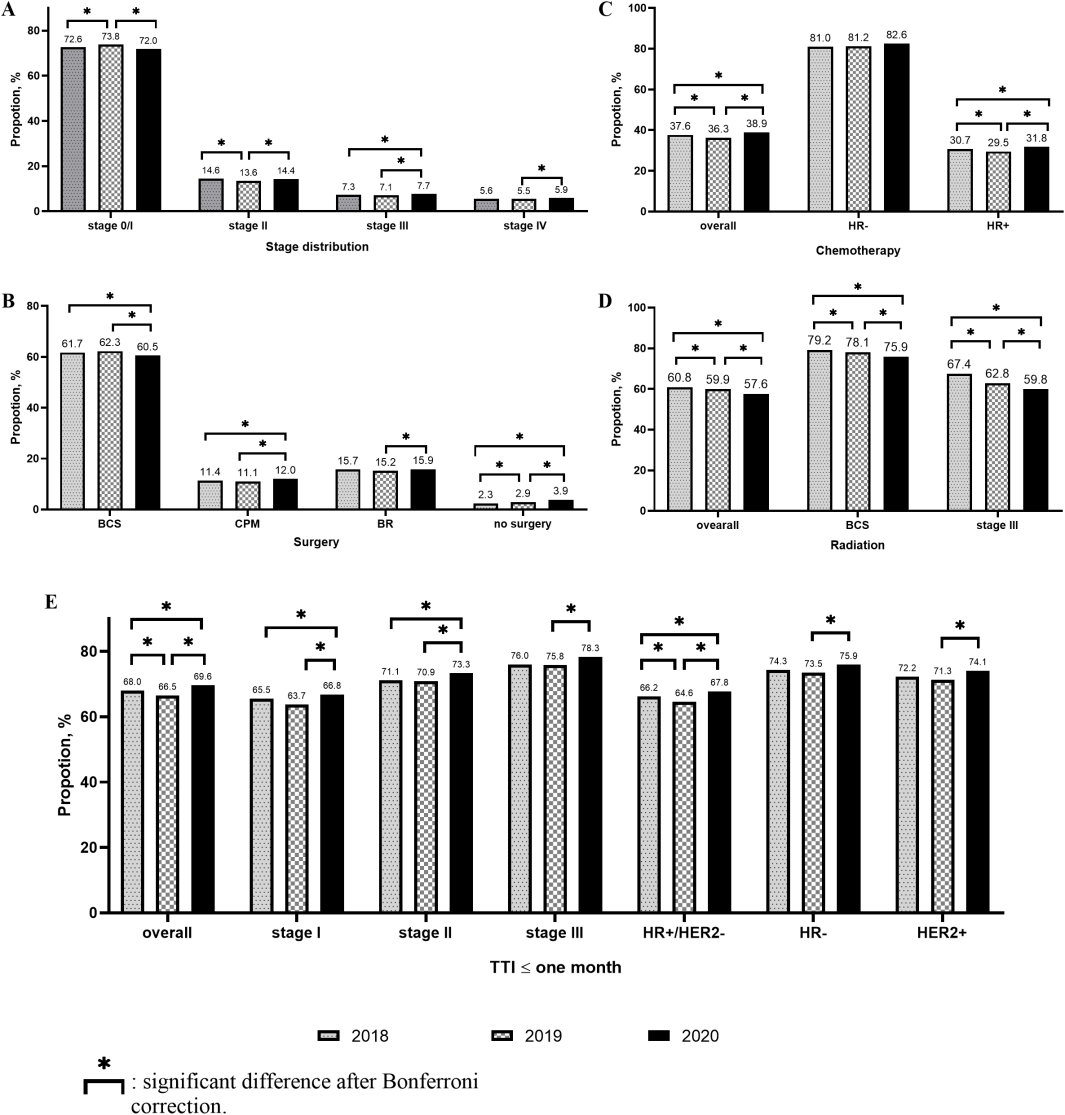
Changes of stage distribution and treatment received for early breast cancer. (**A**. Stage distribution; **(B)** Surgery; **(C)** Chemotherapy; **(D)** Radiation; **(E)** Time to treatment initiation (TTI) ≤ one month.

**Figure 3:**
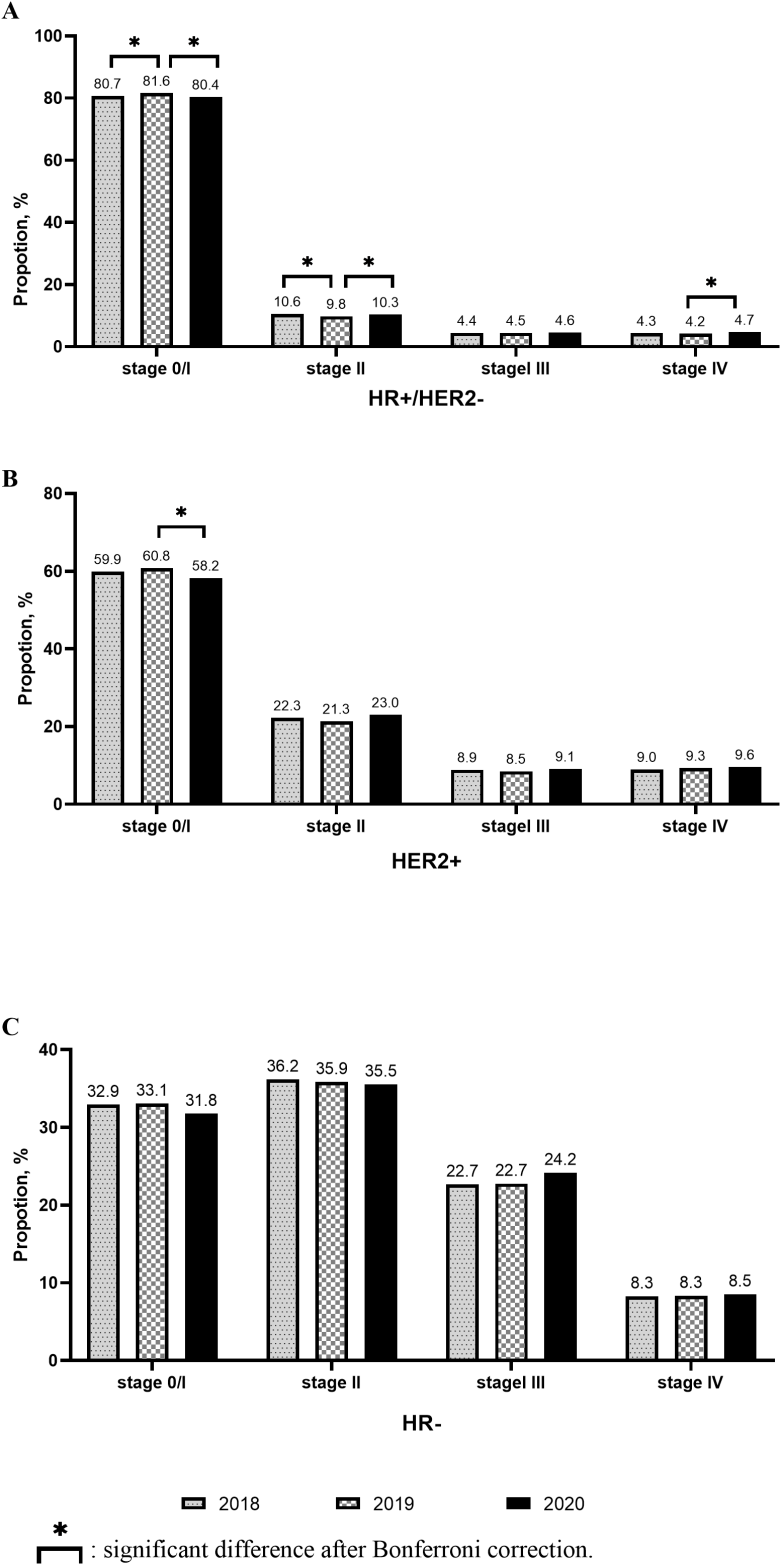
Changes of stage distribution in different subtypes.

Compared to 2018, the proportion of Stage 0–I breast cancer patients increased, while that of Stage II breast cancer patients decreased in 2019. Meanwhile, no significant change was observed in the proportion of Stage III–IV breast cancer patients between 2018 and 2019. However, in 2020, the proportion of Stage 0–I breast cancer patients decreased significantly, while that of Stage II–IV breast cancer patients increased significantly (**Figure 2A**). Similar results were observed in the hormone receptor (HR) positive (+)/human epidermal growth factor receptor (HER) 2 negative (−) populations (**Figure 3A**). However, only the HER2+ patients showed a statistically significant reduction in the proportion of Stage 0–I breast cancer patients in 2020 (**Figure 3B**). In patients with aggressive subtypes of breast cancer, i.e., HER2+ or HR− breast cancer, no significant change was observed in the proportion of patients with Stage III–IV breast cancer (**Figures 3B, C**).

### 3.2 Changes in treatment modalities for early breast cancer

In early breast cancer patients, the proportion of patients without surgery increased significantly. The proportion of breast-conserving surgery (BCS) cases decreased significantly in 2020 than in 2018 or 2019. The proportion of contralateral prophylactic mastectomy (CPM) and immediate breast reconstruction (IBR) cases increased significantly in 2020 than in 2019 (**Figure 2B**).

**Figure 4:**
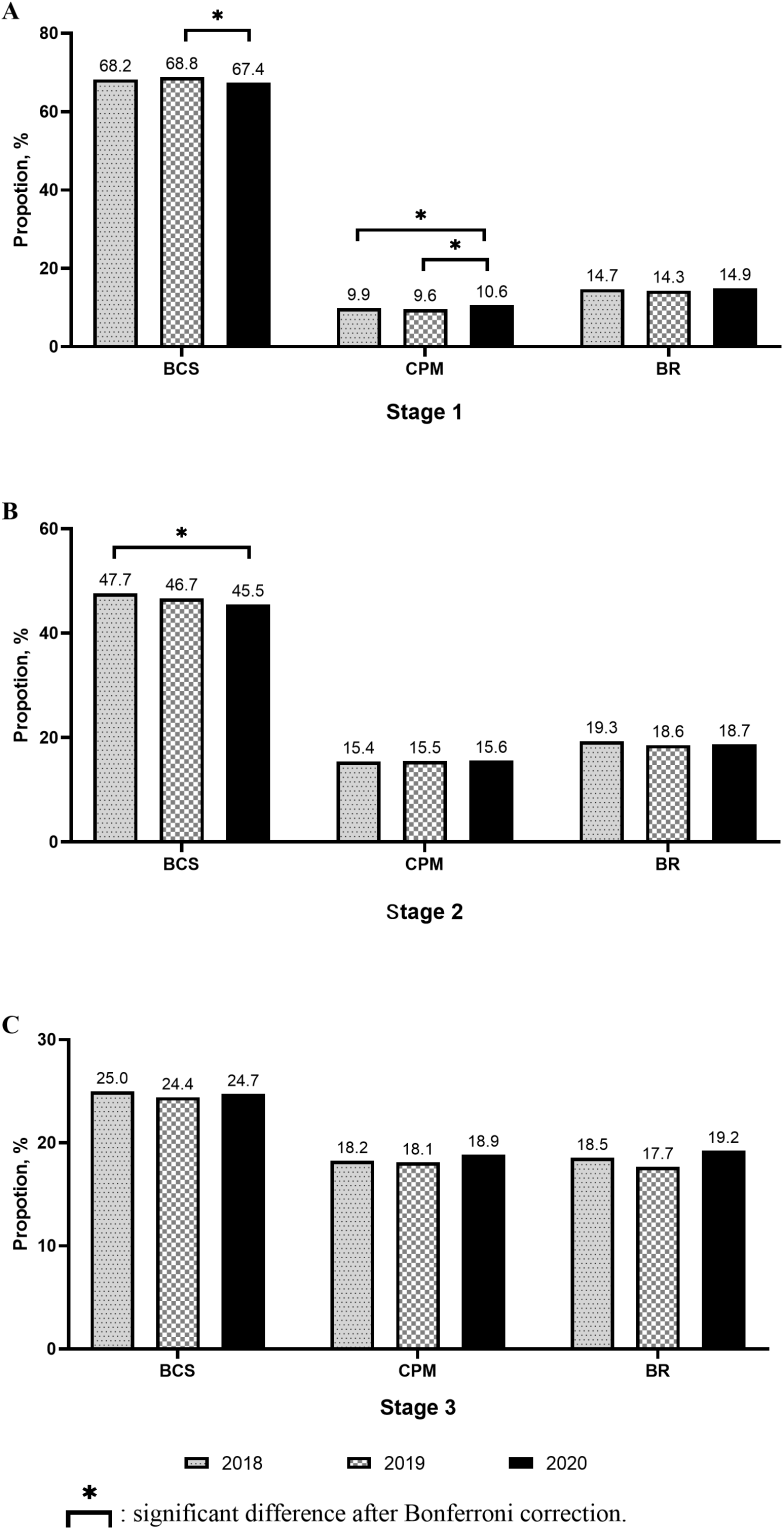
Changes of surgery received in different stages.

In Stage I breast cancer patients, the proportion of BCS cases decreased in 2020 compared to that in 2019, while that of CPM cases increased significantly compared to that in 2018 or 2019. The proportion of IBR cases did not change significantly (**Figure 4A**). In Stage II or III breast cancer patients, no significant change was observed in the proportion of BCS, CPM, and IBR cases among the three cohorts (**Figures 4B, C**).

Although the proportion of chemotherapy cases decreased significantly in 2019, it was significantly higher in 2020 than in 2018 or 2019. The same was true among patients with HR+ breast cancer. However, among HR− patients, the proportion of chemotherapy cases did not differ significantly among these three cohorts. In HR+/HER2− Stage I breast cancer patients, the increased proportion of chemotherapy cases was statistically significant in 2020 (**Figure 2C**). The proportion of patients undergoing radiation decreased significantly every year, as observed in patients undergoing BCS or those with Stage III breast cancer (**Figure 2D**).

### 3.3 Treatment delay in early breast cancer

**Figure 5:**
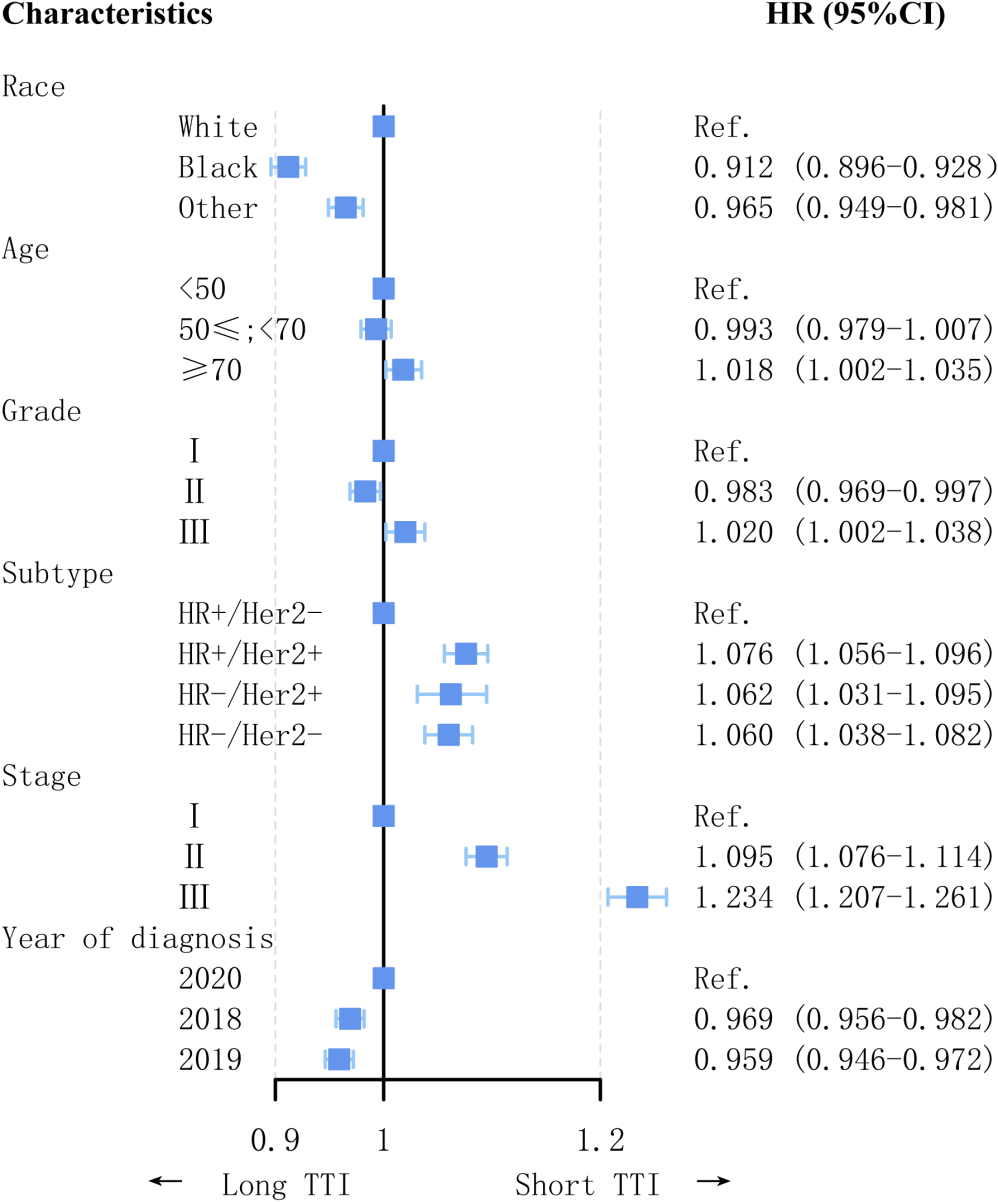
Factors associated with time to treatment initiation (TTI).

The proportion of patients with TTI within one month was the lowest in 2019 and highest in 2020. In all patient subgroups, the proportion of patients with TTI within one month was significantly higher in 2020 than in 2019 (**Figure 2E**). Factors associated with TTI are shown in **Figure 5**. Patients diagnosed during the COVID-19 pandemic had a shorter TTI than those diagnosed in the pre-pandemic period (2018: HR = 0.969, 95% CI = 0.956–0.982, p < 0.001; 2019: HR = 0.959, 95% CI = 0.946–0.972, p < 0.001). Factors associated with short TTI included histological tumor Grade 3 (HR = 1.020, 95% CI = 1.002–1.038, p = 0.033), invasive subtype (HR+/HER2+: HR = 1.076, 95% CI = 1.056–1.096, p < 0.001; HR−/HER2+: HR = 1.062, 95% CI = 1.031– 1.095, p < 0.001; HR−/HER2−: HR = 1.060, 95% CI = 1.038–1.082, p < 0.001), higher cancer stage (Stage II: HR = 1.095, 95% CI = 1.076–1.114, p < 0.001; Stage III: HR = 1.234, 95% CI = 1.207–1.261, p < 0.001), and age >70 years (HR = 1.018, 95% CI = 1.002–1.035, p = 0.023). White patients had a significantly shorter TTI than non-White patients (Blacks: HR = 0.912, 95% CI = 0.896–0.928, p < 0.001; Other: HR = 0.965, 95% CI = 0.949–0.981, p < 0.001).

### 3.4 Year of diagnosis and breast cancer survival in early breast cancer

There were 251 deaths in the 2020 cohort, of which 94 deaths were due to breast cancer. A multivariable Cox proportional hazards model was used to assess the factors associated with OS and BCSS (**Table 2**). The OS (HR = 0.887, 95% CI = 0.766–1.026, p = 0.105) and BCSS (HR = 0.998, 95% CI = 0.789–1.262, p = 0.985) did not differ significantly between 2019 and 2020. However, compared to diagnosis in 2020, diagnosis in 2018 was an independent factor associated with better OS (HR = 0.861, 95% CI = 0.743–0.996, p = 0.045) but not better BCSS (HR = 1.001, 95% CI = 0.791–1.267, p = 0.994) (**Tables 2**, **3**). Results for this modified Cox model were similar between 2018 and 2020 (OS: HR = 0.842, 95% CI = 0.723–0.981, p = 0.027; BCSS: HR = 0.945, 95% CI = 0.74–1.208, p = 0.652) (**Tables 2**, **4**). In the population receiving chemotherapy or radiation, the year of diagnosis was not an independent factor associated with survival. However, in the patients who did not receive chemotherapy, the year of diagnosis significantly affected the OS (HR = 0.814, 95% CI = 0.675–0.982, p = 0.031) (**Table 2**).

**Table 2:** The impact of year of diagnosis on survival among patients with early breast cancer.

		Overall survival					Breast cancer-sepcific survival				
		95%CI					95%CI				
		Events	HR	Low	High	*P*	Events	HR	Low	High	*P*
All patients	2020	251	Reference				94	Reference			
	2018	2056	0.861	0.743	0.996	0.045	944	1.001	0.791	1.267	0.994
	2019	1117	0.887	0.766	1.026	0.105	503	0.998	0.789	1.262	0.985
All patients (modify)	2020	251	Reference				94	Reference			
	2018	596	0.842	0.723	0.981	0.027	254	0.945	0.74	1.208	0.652
	2019	677	0.912	0.786	1.06	0.231	299	1.055	0.83	1.341	0.663
Subgroup											
Patients with chemotherapy	2020	97	Reference				40	Reference			
	2018	863	0.933	0.738	1.181	0.566	593	1.28	0.902	1.816	0.166
	2019	447	0.908	0.718	1.148	0.418	295	1.226	0.865	1.739	0.252
Patients without chemotherapy	2020	154	Reference				54	Reference			
	2018	1193	0.814	0.675	0.982	0.031	351	0.757	0.548	1.045	0.09
	2019	670	0.873	0.725	1.052	0.153	208	0.805	0.584	1.109	0.185
Patients with radiation	2020	35	Reference				12	Reference			
	2018	763	0.793	0.547	1.151	0.222	159	0.978	0.525	1.822	0.944
	2019	330	0.857	0.591	1.242	0.414	414	0.929	0.498	1.733	0.818
Patients without radiation	2020	216	Reference				82	Reference			
	2018	1293	0.893	0.761	1.049	0.167	530	1.018	0.788	1.317	0.889
	2019	787	0.899	0.767	1.055	0.192	344	1.037	0.803	1.338	0.782

**Table 3:** Factors associated with OS and BCSS in a Cox proportional hazards model of patients diagnosed with early breast cancer in 2018,2019, and 2020.

Characteristics		OS				BCSS			
		95% CI				95% CI			
		HR	Low	High	*P*	HR	Low	High	*P*
Race									
	White	Reference				Reference			
	Black	1.312	1.197	1.438	<0.001	1.174	1.029	1.339	0.017
	Other	0.696	0.611	0.793	<0.001	0.743	0.617	0.894	0.002
Age									
	<50	Reference				Reference			
	50≤;<70	1.531	1.363	1.72	<0.001	1.179	1.024	1.358	0.022
	≥70	4.048	3.606	4.544	<0.001	2.37	2.045	2.748	<0.001
Grade									
	I	Reference				Reference			
	II	1.161	1.042	1.293	0.007	1.768	1.385	2.257	<0.001
	III	1.595	1.412	1.802	<0.001	2.974	2.313	3.824	<0.001
Subtype									
	HR+/Her2-	Reference				Reference			
	HR+/Her2+	0.987	0.866	1.126	0.848	0.781	0.634	0.962	0.02
	HR-/Her2+	1.152	0.984	1.348	0.078	1.013	0.816	1.256	0.91
	HR-/Her2-	1.444	1.312	1.59	<0.001	1.555	1.371	1.764	<0.001
Stage									
	I	Reference				Reference			
	II	2.472	2.241	2.727	<0.001	4.366	3.714	5.133	<0.001
	III	7.348	6.683	8.078	<0.001	17.644	15.16	20.536	<0.001
Radiation									
	No	Reference				Reference			
	Yes	0.341	0.317	0.367	<0.001	0.366	0.329	0.407	<0.001
Chemotherapy									
	No	Reference				Reference			
	Yes	0.773	0.708	0.844	<0.001	0.935	0.822	1.063	0.305
Year of diagnosis									
	2020	Reference				Reference			
	2018	0.861	0.743	0.996	0.045	1.001	0.791	1.267	0.994
	2019	0.887	0.766	1.026	0.105	0.998	0.789	1.262	0.985

**Table 4:** Factors associated with OS and BCSS in a modified Cox proportional hazards model of patients diagnosed with early breast cancer in 2018, 2019, and 2020.

Characteristics		OS				BCSS			
		95% CI				95% CI			
		HR	Low	High	*P*	HR	Low	High	*P*
Race									
	White	Reference				Reference			
	Black	1.381	1.207	1.58	<0.001	1.33	1.093	1.618	0.004
	Other	0.683	0.561	0.833	<0.001	0.719	0.535	0.966	0.028
Age									
	<50	Reference				Reference			
	50≤;<70	2.032	1.675	2.464	<0.001	1.443	1.13	1.842	0.003
	≥70	5.251	4.338	6.357	<0.001	3.443	2.693	4.401	<0.001
Grade									
	I	Reference				Reference			
	II	1.185	1.004	1.397	0.045	1.766	1.215	2.565	0.003
	III	1.557	1.294	1.875	<0.001	2.666	1.814	3.919	<0.001
Subtype									
	HR+/Her2-	Reference				Reference			
	HR+/Her2+	1.326	1.105	1.592	0.002	0.987	0.729	1.337	0.933
	HR-/Her2+	1.365	1.089	1.712	0.007	1.107	0.797	1.538	0.544
	HR-/Her2-	1.555	1.345	1.799	<0.001	1.544	1.269	1.88	<0.001
Stage									
	I	Reference				Reference			
	II	2.48	2.145	2.868	<0.001	4.729	3.687	6.066	<0.001
	III	6.789	5.894	7.819	<0.001	18.161	14.376	22.942	<0.001
Radiation									
	No	Reference				Reference			
	Yes	0.199	0.175	0.225	<0.001	0.191	0.158	0.231	<0.001
Chemotherapy									
	No	Reference				Reference			
	Yes	0.772	0.678	0.879	<0.001	0.943	0.78	1.141	0.546
Year of diagnosis									
	2020	Reference				Reference			
	2018	0.842	0.723	0.981	0.027	0.945	0.74	1.208	0.652
	2019	0.912	0.786	1.06	0.231	1.055	0.83	1.341	0.663

## 4 Discussion

This study found a decline in the number of breast cancer diagnoses during the pandemic in 2020 compared to that in 2018 or 2019. The proportion of Stage 0–I patients decreased in 2020 compared to 2019. Moreover, the proportion of patients undergoing radiation and BCS decreased in 2020, while that of patients undergoing chemotherapy increased. Patients diagnosed in 2020 experienced significantly fewer treatment delays compared to those diagnosed in 2018 or 2019. However, diagnosis of breast cancer in 2020 emerged as a significant independent risk factor for OS in breast cancer patients.

We observed that the number of patients diagnosed with and treated for breast cancer in 2020 was the lowest. Since the Dutch National Breast Screening Program was suspended by the COVID-19 pandemic starting in Week 12 of 2020, the incidence of screen-detected tumors nearly dropped to zero during weeks 14–25 (5). In the United States, a marked decrease of 54.9% in breast cancer incidence rates was observed during the pandemic (14). Some single-institution studies from different countries reported that patients presented with more advanced stages of breast cancer in 2020 (10, 11, 15). Similarly, we observed an increase in the proportion of stage III or IV breast cancers increased in 2020. However, the population-based study from the Netherlands Cancer Registry did not find a shift towards higher stage breast cancers (5). This discrepancy could be attributed to several reasons. Firstly, the time point of the Dutch study cut-off was August 2020. The breast cancer screening program restarted at a reduced capacity, and there were some delayed breast cancer diagnoses that have not yet been discovered. Secondly, the duration of disruptions to breast screening varied across countries. Poelhekken et al. used the simulation model on radiation risk and breast cancer screening and found no clinically significant changes in screening-detected tumor sizes after a 3-month interruption. However, interruptions of 6 or 12 months considerably influenced the tumor size (2).Our observations differed from the population study from Brazilian, wherein the HR negative or HER2 positive breast cancer, regarded as aggressive tumor subtypes, did not show higher percentage of patients with advanced-stage disease during the pandemic (16).

The Pandemic Breast Cancer Consortium provided expert guidance to categorize patients with breast cancer into priority levels to ensure timely and reasonable treatment during the COVID-19 pandemic (17). It recommended that patients eligible for breast conservation therapy were advised against undergoing mastectomy to minimize operative duration. Additionally, for those patients scheduled for mastectomy, contralateral prophylactic mastectomy and immediate reconstruction were discouraged to further reduce surgical time and the associated risk of complications. The COVID-19 pandemic affected surgical activities, but in contrast to these recommendations. In Italy, the mastectomy–BCS ratio was 39–61% in 2019 and 42–58% in 2020. Mastectomies with direct to implant reconstruction surgeries increased by 15%, whereas mastectomies with immediate expander reconstruction decreased by 20% (18). A study based on the Specific Health Checkups program in Japan showed that the proportion of BCS cases without axillary lymph node dissection decreased significantly, whereas no decrease was observed for other surgery types (19). All these studies, including ours, demonstrated that the proportion of patients not undergoing surgery increased significantly in 2020. The proportion of BCS cases decreased in 2020 compared to 2018 or 2019. This was also true for Stage I breast cancer patients. In light of the uncertainty caused by the pandemic, the possibility of missed or delayed radiotherapy, and frequent hospital visits for radiotherapy, some of these patients decided on mastectomy. We also found a significant increase in the proportion of CPM and IBR cases in the overall population. Patients refusing BCS were more likely to resort to IBR for esthetic breast appearance. Decreased surgical volume and low-priority surgery for non-tumor diseases led to more spare plastic surgeons than in the pre-pandemic period. Furthermore, poor communication between doctors and patients during the pandemic could have contributed to the change in surgical approach. Decisions for CPM appear to be patient-driven for reasons such as genetic testing, strong family cancer history, and increased concern about cancer recurrence. However, the knowledge of and recommendations for CPM among women with breast cancer in the United States are limited (20). Therefore, CPM is prevalent among patients without clinical indications. Physician–patient communication regarding CPM can effectively reduce potential overtreatment (21).

The proportion of radiation cases decreased significantly in patients with Stage III or those undergoing BCS, which are absolute indications for radiation as per the recommended guidelines. This could be attributed to several reasons. First, recently, some guidelines recommend considering the omission of post-lumpectomy radiation in older breast cancer patients (22). Second, some patients may refuse or delay radiation therapy to decrease the chance of COVID-19 infection. Third, delayed radiation therapy was widespread during the pandemic, which may have affected the data entry accuracy and timeliness. Finally, we cannot exclude the presence of undertreatment. In addition, practical patterns of radiotherapy to early breast cancer have changed. International guidelines recommended that ultra-hypofractionated radiotherapy was preferentially used in patients with lower risk disease in order to minimize patient time spent in the hospital (23). A population-based study from UK found the use of hypofractionated regimens rapidly increased by 60·4% in 2020 (24). MD Anderson Cancer Center reported the percentage of ultra-hypofractionated radiotherapy increased from 4.3% in March-April 2020 to 45.5% in July-August 2020 (25).

The application of genomic tests greatly reduced the chemotherapy application. 21-gene RS testing and 70-gene signature could aid to avoid adjuvant chemotherapy (26, 27). We found the proportion of chemotherapy cases decreased in 2019. However, it rose rapidly in 2020, even surpassing that in 2018. These results corroborate the findings of a population-based cohort study from the Netherlands and Canada (28, 29). In some studies from the United States, chemotherapy cases had increased in 2020 (30, 31). This was partly due to the advanced stages of breast cancer cases diagnosed in 2020. Additionally, the increase in the proportion of neoadjuvant chemotherapy cases could explain it. It is important to note that the proportion of patients treated with chemotherapy has significantly increased among those with Stage I HR+/HER2- tumors. However, this differs from the actual daily clinical practice. There may be overtreatment in these patients. Data obtained from the National Cancer Database (NCDB) indicated a statistically significant decrease in the proportion of patients undergoing BCS and radiotherapy, as reported in our study. However, no statistically significant change was observed in the proportion of patients receiving radiation or systemic therapy among those diagnosed with Stages II–IV breast cancer (32). The NCDB is a hospital-based and not a population-based cancer registry. Treatment modalities commonly provided in outpatient settings may be missed, such as chemotherapy.

Shorter TTI was associated with lower mortality and higher accessibility to cancer therapy (33, 34). Our study found that the proportion of patients with TTI less than one month increased significantly in 2020. The time-to-event analysis showed that patients received treatment faster in 2020. An NCDB study showed that the COVID-19 pandemic was not associated with delay in systemic therapy (35). The time between diagnosis and treatment initiation decreased in patients diagnosed with breast cancer even during lockdown in the Dutch population (29). Among the patients diagnosed with COVID-19, those with breast cancer received treatment sooner than those with other tumors. This could be attributed to a decrease in the incidence of breast cancer, application of neoadjuvant treatment (36), and prioritization for a different surgery (17). Neoadjuvant therapy was used for safe postponement of surgery in selected cases, which is recommended by several guidelines (17, 37–39). Particularly, an increased application of neoadjuvant endocrine therapy was observed (12, 40). Our study revealed that racial minorities, particularly black women, experienced prolonged TTI compared to white individuals. Existing literature consistently shows disparities in TTI for breast cancer patients (41). The COVID-19 pandemic has the potential to worsen existing delays in treatment initiation for black women with breast cancer (42, 43). Further research is necessary to examine the effects of public health emergencies on racial and ethnic disparities in cancer care.

A recent review showed evidence of an increased risk of COVID-19-related death in patients recently diagnosed with cancer, and the risk decreased with time since diagnosis (44). We found that the diagnosis of breast cancer in the year 2020 was a risk factor for OS compared to the diagnosis in 2018; however, the COVID-19 outbreak did not significantly affect the BCSS. This was because of worse OS due to non-breast cancer-related mortality. As mentioned in a study from the United States National Vital Statistics System, the incidence of cancer-related death increased slightly from 2018 to 2021 but was relatively less than the increase in the number of deaths from any cause, indicating that an excess number of persons with cancer died from COVID-19 and other diseases (45). Among patients receiving chemotherapy or radiation, OS did not change during pandemic. In a prospective cohort study, chemotherapy or other anticancer treatment did not increase risk of mortality from COVID-19 disease (46). Some studies on breast cancer showed no association between systemic therapy or loco-regional therapy and increased mortality risk (47, 48). In these studies, older age and pre-existing comorbidities were associated with adverse COVID-19 outcomes in breast cancer patients. These patients were likely to refuse chemotherapy and end up in the non-chemotherapy group during the pandemic. This could be the reason why the pandemic seemed to affect the OS in the non-chemotherapy population.

The pandemic’s impact on the duration of time between cancer diagnosis and initiation of treatment indicated a notable level of resilience in the US (49). Nevertheless, the present study also highlighted significant concerns, encompassing delayed diagnoses and potential deviations from treatment guidelines, such as overuse of chemotherapy, underuse of radiotherapy and underuse of BCS. Evidently, the pandemic greatly influenced the treatment approaches for early-stage breast cancer.

The pandemic has worsened pre-existing issues like poor doctor-patient communication and unequal healthcare access. Chen et al. showed that telehealth can improve communication and increase the effectiveness of cancer screening during this time (50). It would be helpful to ensure timely diagnosis and optimal treatment for patients with breast cancer in future public health emergencies.

This study has several limitations. First, it was a retrospective analysis with a short follow-up period, necessitating further studies with a longer follow-up duration. Second, the SEER database lacked detailed information regarding the specific month of diagnosis in 2020, and we had to consider the entire year of 2020 for comparison with 2018 or 2019. Third, we were unable to examine the status of comorbidities, COVID-19, and insurance as possible confounders in the survival analysis, as we lacked information on them.

## 5 Conclusion

To the best of our knowledge, this is the first nationwide cohort study to investigate the effect of the COVID-19 pandemic on the presentation, treatment, and survival of early breast cancer patients. We observed a shift towards advanced-stage breast cancers and a potential for over- or undertreatment in 2020. Patients with breast cancer had worse OS during the pandemic compared to that in the pre-pandemic period; however, their BCSS remained unaffected. Further research is necessary to investigate the reasons behind the divergence of clinical practice from established guidelines and evaluate the impact of such deviations on breast cancer outcomes.

## Data Availability

The original contributions presented in the study are included in the article/supplementary material. Further inquiries can be directed to the corresponding author.

## References

[1] Nokhostin F Dargahi M Tutunchi S Rezaeeyan H. Evaluation of prognostic/diagnostic value of hematological markers in the detection of inflammation in coronavirus disease: A review study. J Adv Med Biomed Res. (2020) 28:171–4. doi: 10.30699/jambs.28.128.171

[2] Poelhekken K Greuter MJW de Munck L Siesling S Brokken FB de Bock GH. Long-term effects of the interruption of the Dutch breast cancer screening program due to COVID-19: A modelling study. Prev Med. (2023) 166:107376. doi: 10.1016/j.ypmed.2022.107376 36493865 PMC9722618

[3] Lee R Xu W Dozier M McQuillan R Theodoratou E Figueroa J. A rapid review of COVID-19’s global impact on breast cancer screening participation rates and volumes from January to December 2020. Elife. (2023) 12. doi: 10.7554/eLife.85680 PMC10569787 37698273

[4] Bakouny Z Paciotti M Schmidt AL Lipsitz SR Choueiri TK Trinh QD. Cancer screening tests and cancer diagnoses during the COVID-19 pandemic. JAMA Oncol. (2021) 7:458–60. doi: 10.1001/jamaoncol.2020.7600 PMC7809614 33443549

[5] Eijkelboom AH de Munck L Lobbes MBI van Gils CH Wesseling J Westenend PJ. Impact of the suspension and restart of the Dutch breast cancer screening program on breast cancer incidence and stage during the COVID-19 pandemic. Prev Med. (2021) 151:106602. doi: 10.1016/j.ypmed.2021.106602 34217417 PMC9755636

[6] Gathani T Dodwell D Horgan K. The impact of the first 2 years of the COVID-19 pandemic on breast cancer diagnoses: a population-based study in England. Br J Cancer. (2023) 128:481–3. doi: 10.1038/s41416-022-02054-4 PMC9660015 36371502

[7] Kaufman HW Chen Z Niles J Fesko Y. Changes in the number of US patients with newly identified cancer before and during the coronavirus disease 2019 (COVID-19) pandemic. JAMA Netw Open. (2020) 3:e2017267. doi: 10.1001/jamanetworkopen.2020.17267 32749465 PMC7403918

[8] DeGroff A Miller J Sharma K Sun J Helsel W Kammerer W. COVID-19 impact on screening test volume through the National Breast and Cervical Cancer early detection program, January-June 2020, in the United States. Prev Med. (2021) 151:106559. doi: 10.1016/j.ypmed.2021.106559 34217410 PMC9026719

[9] Schommer L Mikulski MF Goodgame B Brown KM. Racial disparities in breast cancer presentation and diagnosis in COVID-era central texas. J Surg Res. (2023) 288:79–86. doi: 10.1016/j.jss.2023.02.021 36948036 PMC10026721

[10] Borsky K Shah K Cunnick G Tsang-Wright F. Pattern of breast cancer presentation during the COVID-19 pandemic: results from a cohort study in the UK. Future Oncol. (2022) 18:437–43. doi: 10.2217/fon-2021-0970 PMC8763213 35018787

[11] Bonadio RC Messias AP Moreira OA Leis LV Orsi BZ Testa L. Impact of the COVID-19 pandemic on breast and cervical cancer stage at diagnosis in Brazil. Ecancermedicalscience. (2021) 15:1299. doi: 10.3332/ecancer.2021.1299 34824622 PMC8580713

[12] Tonneson JE Hoskin TL Day CN Durgan DM Dilaveri CA Boughey JC. Impact of the COVID-19 pandemic on breast cancer stage at diagnosis, presentation, and patient management. Ann Surg Oncol. (2022) 29:2231–9. doi: 10.1245/s10434-021-11088-6 PMC8609838 34812981

[13] Mathew G Agha R Albrecht J Goel P Mukherjee I Pai P. STROCSS 2021: Strengthening the reporting of cohort, cross-sectional and case-control studies in surgery. Int J Surg. (2021) 96:106165. doi: 10.1016/j.ijsu.2021.106165 34774726

[14] Howlader N Bhattacharya M Scoppa S Miller D Noone AM Negoita S. Cancer and COVID-19: U.S. Cancer incidence rates during the first year of the pandemic. J Natl Cancer Inst. (2023). doi: 10.1093/jnci/djad205 PMC10852612 37796818

[15] Adachi K Kimura F Takahashi H Kaise H Yamada K Ueno E. Delayed diagnosis and prognostic impact of breast cancer during the COVID-19 pandemic. Clin Breast Cancer. (2023) 23:265–71. doi: 10.1016/j.clbc.2023.01.001 PMC9829603 36717319

[16] Resende CAA Fernandes Cruz HM Costa ESM Paes RD Dienstmann R Barrios CHE. Impact of the COVID-19 pandemic on cancer staging: an analysis of patients with breast cancer from a community practice in Brazil. JCO Glob Oncol. (2022) 8:e2200289. doi: 10.1200/GO.22.00289 36351212 PMC10166498

[17] Dietz JR Moran MS Isakoff SJ Kurtzman SH Willey SC Burstein HJ. Recommendations for prioritization, treatment, and triage of breast cancer patients during the COVID-19 pandemic. the COVID-19 pandemic breast cancer consortium. Breast Cancer Res Treat. (2020) 181:487–97. doi: 10.1007/s10549-020-05644-z PMC7181102 32333293

[18] Sgarzani R Macrì G Gurrado A Curcio A De Lorenzi F Galimberti V. The impact of COVID-19 pandemic on breast surgery in Italy: a multi-centric retrospective observational study. Updates Surg. (2023) 75:735–41. doi: 10.1007/s13304-023-01474-y PMC9987352 36877431

[19] Fujita M Hashimoto H Nagashima K Suzuki K Kasai T Yamaguchi K. Impact of coronavirus disease 2019 pandemic on breast cancer surgery using the National Database of Japan. Sci Rep. (2023) 13:4977. doi: 10.1038/s41598-023-32317-w 36973536 PMC10041497

[20] Hawley ST Jagsi R Morrow M Janz NK Hamilton A Graff JJ. Social and clinical determinants of contralateral prophylactic mastectomy. JAMA Surg. (2014) 149:582–9. doi: 10.1001/jamasurg.2013.5689 PMC4703398 24849045

[21] Jagsi R Hawley ST Griffith KA Janz NK Kurian AW Ward KC. Contralateral prophylactic mastectomy decisions in a population-based sample of patients with early-stage breast cancer. JAMA Surg. (2017) 152:274–82. doi: 10.1001/jamasurg.2016.4749 PMC5531287 28002555

[22] Knowlton CA. Breast cancer management during the COVID-19 pandemic: the radiation oncology perspective. Curr Breast Cancer Rep. (2022) 14:8–16. doi: 10.1007/s12609-022-00441-7 35251487 PMC8881209

[23] Coles CE Aristei C Bliss J Boersma L Brunt AM Chatterjee S. International guidelines on radiation therapy for breast cancer during the COVID-19 pandemic. Clin Oncol (Royal Coll Radiol (Great Britain)). (2020) 32:279–81. doi: 10.1016/j.clon.2020.03.006 PMC7270774 32241520

[24] Spencer K Jones CM Girdler R Roe C Sharpe M Lawton S. The impact of the COVID-19 pandemic on radiotherapy services in England, UK: a population-based study. Lancet Oncol. (2021) 22:309–20. doi: 10.1016/S1470-2045(20)30743-9 PMC7825861 33493433

[25] Corrigan KL Lei X Ahmad N Arzu I Bloom E Chun SG. Adoption of ultrahypofractionated radiation therapy in patients with breast cancer. Adv Radiat Oncol. (2022) 7:100877. doi: 10.1016/j.adro.2021.100877 35387420 PMC8977907

[26] Piccart M van ‘t Veer LJ Poncet C Lopes Cardozo JMN Delaloge S Pierga JY. 70-gene signature as an aid for treatment decisions in early breast cancer: updated results of the phase 3 randomised MINDACT trial with an exploratory analysis by age. Lancet Oncol. (2021) 22:476–88. doi: 10.1016/S1470-2045(21)00007-3 33721561

[27] Sparano JA Gray RJ Makower DF Pritchard KI Albain KS Hayes DF. Adjuvant chemotherapy guided by a 21-gene expression assay in breast cancer. New Engl J Med. (2018) 379:111–21. doi: 10.1056/NEJMoa1804710 PMC6172658 29860917

[28] Habbous S Tai X Beca JM Arias J Raphael MJ Parmar A. Comparison of use of neoadjuvant systemic treatment for breast cancer and short-term outcomes before vs during the COVID-19 era in ontario, Canada. JAMA Netw Open. (2022) 5:e2225118. doi: 10.1001/jamanetworkopen.2022.25118 35917122 PMC9346546

[29] Eijkelboom AH de Munck L Menke-van der Houven van Oordt CW Broeders MJM van den Bongard D Strobbe LJA. Changes in breast cancer treatment during the COVID-19 pandemic: a Dutch population-based study. Breast Cancer Res Treat. (2023) 197:161–75. doi: 10.1007/s10549-022-06732-y PMC9638417 36334188

[30] Milgrom ZZ Milgrom DP Han Y Hui SL Haggstrom DA Fisher CS. Breast cancer screening, diagnosis, and surgery during the pre- and peri-pandemic: experience of patients in a statewide health information exchange. Ann Surg Oncol. (2023) 30:2883–94. doi: 10.1245/s10434-023-13183-2 PMC9904246 36749504

[31] Malmgren JA Guo B Atwood MK Hallam P Roberts LA Kaplan HG. COVID-19 related change in breast cancer diagnosis, stage, treatment, and case volume: 2019-2021. Breast Cancer Res Treat. (2023) 202:105–15. doi: 10.1007/s10549-023-06962-8 PMC10504101 37584882

[32] Nogueira LM Schafer EJ Fan Q Wagle NS Zhao J Shi KS. Assessment of changes in cancer treatment during the first year of the COVID-19 pandemic in the US. JAMA Oncol. (2023). doi: 10.1001/jamaoncol.2023.4513 PMC10636648 37943539

[33] Cone EB Marchese M Paciotti M Nguyen DD Nabi J Cole AP. Assessment of time-to-treatment initiation and survival in a cohort of patients with common cancers. JAMA network Open. (2020) 3:e2030072. doi: 10.1001/jamanetworkopen.2020.30072 33315115 PMC7737088

[34] Nguyen DD Paciotti M Marchese M Cole AP Cone EB Kibel AS. Effect of medicaid expansion on receipt of definitive treatment and time to treatment initiation by racial and ethnic minorities and at minority-serving hospitals: A patient-level and facility-level analysis of breast, colon, lung, and prostate cancer. JCO Oncol practice. (2021) 17:e654–65. doi: 10.1200/OP.21.00010 33974827

[35] Chung SH Romatoski KS Rasic G Beaulieu-Jones BR Kenzik K Merrill AL. Impact of the COVID-19 pandemic on delays to breast cancer surgery: ripples or waves? Ann Surg Oncol. (2023) 30:6093–103. doi: 10.1245/s10434-023-13878-6 37526751

[36] Sheng JY Santa-Maria CA Mangini N Norman H Couzi R Nunes R. Management of breast cancer during the COVID-19 pandemic: A stage- and subtype-specific approach. JCO Oncol Pract. (2020) 16:665–74. doi: 10.1200/OP.20.00364 PMC7564133 32603252

[37] Manoj Gowda S Kabeer KK Jafferbhoy S Marla S Soumian S Misra V. Breast cancer management guidelines during COVID-19 pandemic. Indian J Surg. (2020) 82:251–8. doi: 10.1007/s12262-020-02466-7 PMC7329358 32837081

[38] Martin M Guerrero-Zotano A Montero Á Jara C Filipovich E Rojo F. GEICAM guidelines for the management of patients with breast cancer during the COVID-19 pandemic in Spain. Oncologist. (2020) 25:e1339–45. doi: 10.1634/theoncologist.2020-0363 PMC7405405 32652782

[39] Kawate T Yoshida A Sugae S Asaga S Kaise H Saji S. Recommendations for the management of breast cancer patients during the COVID-19 pandemic from the Japan Breast Cancer Society. Breast Cancer. (2021) 28:247–53. doi: 10.1007/s12282-020-01214-9 PMC7895736 33609229

[40] Wilke LG Nguyen TT Yang Q Hanlon BM Wagner KA Strickland P. Analysis of the impact of the COVID-19 pandemic on the multidisciplinary management of breast cancer: review from the american society of breast surgeons COVID-19 and mastery registries. Ann Surg Oncol. (2021) 28:5535–43. doi: 10.1245/s10434-021-10639-1 PMC8384097 34431019

[41] Fasano GA Bayard S Gillot T Hannibal Z Pedreira M Newman L. Disparities in time to treatment for breast cancer: existing knowledge and future directions in the COVID-19 era. Curr Breast Cancer Rep. (2022) 14:213–21. doi: 10.1007/s12609-022-00469-9 PMC9735127 36530340

[42] Obeng-Gyasi S Oppong B Paskett ED Lustberg M. Purposeful surgical delay and the coronavirus pandemic: how will black breast cancer patients fare? Breast Cancer Res Treat. (2020) 182:527–30. doi: 10.1007/s10549-020-05740-0 PMC7298443 32556796

[43] Patel MI Ferguson JM Castro E Pereira-Estremera C.D Armaiz-Peña G.N Duron Y. Racial and ethnic disparities in cancer care during the COVID-19 pandemic. JAMA network Open. (2022) 5:e2222009. doi: 10.1001/jamanetworkopen.2022.22009 35834248 PMC9284331

[44] Steinberg J Hughes S Hui H Allsop MJ Egger S David M. Risk of COVID-19 death for people with a pre-existing cancer diagnosis prior to COVID-19-vaccination: A systematic review and meta-analysis. Int J Cancer. (2023). doi: 10.1002/ijc.34798 PMC10922788 38083979

[45] Henley SJ Dowling NF Ahmad FB Ellington TD Wu M Richardson LC. COVID-19 and other underlying causes of cancer deaths - United States, january 2018-july 2022. MMWR Morb Mortal Wkly Rep. (2022) 71:1583–8. doi: 10.15585/mmwr.mm7150a3 PMC9762902 36520660

[46] Lee LY Cazier JB Angelis V Arnold R Bisht V Campton NA. COVID-19 mortality in patients with cancer on chemotherapy or other anticancer treatments: a prospective cohort study. Lancet. (2020) 395:1919–26. doi: 10.1016/S0140-6736(20)31173-9 PMC7255715 32473682

[47] Marks DK Budhathoki N Kucharczyk J Fa'ak F D'Abreo N Kwa M. Outcomes of breast cancer patients treated with chemotherapy, biologic therapy, endocrine therapy, or active surveillance during the COVID-19 pandemic. Oncologist. (2022) 27:89–96. doi: 10.1093/oncolo/oyab042 35641208 PMC8895753

[48] Nagaraj G Vinayak S Khaki AR Sun T Kuderer NM Aboulafia DM. Clinical characteristics, racial inequities, and outcomes in patients with breast cancer and COVID-19: A COVID-19 and cancer consortium (CCC19) cohort study. Elife. (2023), 12. doi: 10.7554/eLife.82618.sa2 PMC10637772 37846664

[49] Janczewski LM Cotler J Merkow RP Palis B Nelson H Mullett T. Alterations in cancer treatment during the first year of the COVID-19 pandemic in the US. JAMA Netw Open. (2023) 6:e2340148. doi: 10.1001/jamanetworkopen.2023.40148 37902756 PMC10616721

[50] Chen RC Haynes K Du S Barron J Katz AJ. Association of cancer screening deficit in the United States with the COVID-19 pandemic. JAMA Oncol. (2021) 7:878–84. doi: 10.1001/jamaoncol.2021.0884 PMC8085759 33914015

